# Unicorn: enhancing single-cell Hi-C data with blind super-resolution for 3D genome structure reconstruction

**DOI:** 10.1093/bioinformatics/btaf177

**Published:** 2025-07-15

**Authors:** Mohan Kumar B Chandrashekar, Rohit Menon, Samuel Olowofila, Oluwatosin Oluwadare

**Affiliations:** Department of Computer Science, University of Colorado, Colorado Springs, 1420 Austin Bluffs Parkway, Colorado Springs, CO, 80918, United States; Department of Computer Science, University of Colorado, Colorado Springs, 1420 Austin Bluffs Parkway, Colorado Springs, CO, 80918, United States; Department of Computer Science, University of Colorado, Colorado Springs, 1420 Austin Bluffs Parkway, Colorado Springs, CO, 80918, United States; Department of Computer Science, University of Colorado, Colorado Springs, 1420 Austin Bluffs Parkway, Colorado Springs, CO, 80918, United States; Department of Biomedical Informatics, University of Colorado Anschutz Medical Campus, 13001 East 17th Place, Aurora, CO, 80045, United States

## Abstract

**Motivation:**

Single-cell Hi-C (scHi-C) data provide critical insights into chromatin interactions at individual cell levels, uncovering unique genomic 3D structures. However, scHi-C datasets are characterized by sparsity and noise, complicating efforts to accurately reconstruct high-resolution chromosomal structures. In this study, we present ScUnicorn, a novel blind super-resolution framework for scHi-C data enhancement. ScUnicorn uses an iterative degradation kernel optimization process, unlike traditional super-resolution approaches, which rely on downsampling, predefined degradation ratios, or constant assumptions about the input data to reconstruct high-resolution interaction matrices. Hence, our approach more reliably preserves critical biological patterns and minimizes noise. Additionally, we propose 3DUnicorn, a maximum likelihood algorithm that leverages the enhanced scHi-C data to infer precise 3D chromosomal structures.

**Results:**

Our evaluation demonstrates that ScUnicorn achieves superior performance over the state-of-the-art methods in terms of Peak Signal-to-Noise Ratio, Structural Similarity Index Measure, and GenomeDisco scores. Moreover, 3DUnicorn’s reconstructed structures align closely with experimental 3D-FISH data, underscoring its biological relevance. Together, ScUnicorn and 3DUnicorn provide a robust framework for advancing genomic research by enhancing scHi-C data fidelity and enabling accurate 3D genome structure reconstruction.

**Availability and implementation:**

Unicorn implementation is publicly accessible at https://github.com/OluwadareLab/Unicorn.

## 1 Introduction

Single-cell Hi-C (scHi-C) data provides information regarding chromatin interactions at the individual cell level, unlike bulk Hi-C, which makes average of interactions from many cells (Nagano *et al.* 2013). This data helps to understand the variability in genome distribution across individual cells, revealing the unique 3D structure of the genome in each cell.

3D reconstruction of scHi-C data provides a visual representation of genome organization, revealing specific chromatin interactions and high-level structural details that are crucial for understanding gene regulation and disease mechanisms (Kos *et al.* 2021). By converting chromosome contact interactions into spatial distances, this process allows for the visualization of the 3D genome structure, helping to elucidate chromatin organization and its impact on gene expression and other genomic interactions. Galitsyna and Gelfand (2021) in their work explored methods for processing sparse contact data and emphasized the importance of computational tools for 3D reconstruction. They highlighted the role of techniques such as molecular dynamics simulations, polymer models, and imputation strategies in reconstructing chromatin 3D structures, addressing data sparsity through computational enhancements.

Numerous computational methods have been developed for 3D reconstruction using single-cell Hi-C data (Banecki *et al.* 2024). The Si-C method (Meng *et al.* 2021) imputes super-resolution genome structures from sparse and noisy scHi-C data by using a Bayesian framework with hierarchical optimization, enabling fast and effective reconstruction of high-resolution 3D chromosomal structures. SIMBA3D (Carstens *et al.* 2016) uses a Bayesian multiscale approach to reconstruct 3D chromosome structures from sparse scHi-C data, integrating bulk Hi-C data as prior information. This method addresses data sparsity through regularization and penalties, improving accuracy, while multiscale gradient-based optimization reduces computational complexity. Other methods, such as chemistry- and physics-based algorithms like DPDchrom (Kos *et al.* 2021) and LJ3D (Zha *et al.* 2021), simulate physical interactions to model chromatin conformations. These techniques introduce novel approaches, such as dissipative particle dynamics and the Lennard-Jones potential for 3D structure prediction. Additionally, mathematics-based methods like SCL (Zhu and Wang 2019) utilize lattice-based models and probabilistic simulations to optimize chromatin dynamics from sparse Hi-C data. However, the primary challenge in 3D reconstruction of scHi-C data lies in its sparsity, due to fewer chromatin interactions occurring in each individual cell, and the noise present, which complicates accurate genome structure inference.

To address the sparsity and noise inherent in scHi-C data, traditional computational methods (Table 1) often rely on predefined downsampling (such as 1/16, 1/32, or 1/100), which may not accurately reflect real-world conditions and can compromise biological fidelity. In contrast, our approach uses dynamic degradation, as implemented in Blind Super-Resolution, which adapts to the dataset’s unique characteristics, preserving critical genomic patterns while enhancing resolution. Here, we introduce ScUnicorn, a blind super-resolution algorithm designed specifically for scHi-C data enhancement. Unlike conventional methods, ScUnicorn avoids static assumptions and downsampling, making it better suited for overcoming the challenges posed by scHi-C data. This innovative approach offers new insights into how scHi-C data can be used for advanced processing, ultimately enabling the development of advanced algorithms for studying chromatin organization and its role in gene regulation and disease progression. Additionally, we present 3DUnicorn, which uses the enhanced scHi-C data from ScUnicorn to reconstruct 3D genome structure through a maximum likelihood algorithm (Oluwadare *et al.* 2018). Together, these algorithms provide a comprehensive solution to advance single-cell genomic research by addressing sparsity and enabling 3D structure reconstruction from scHi-C data.

**Table 1.** A side-by-side comparison of Hi-C data enhancement methods.

Method	Publication date	Single cell	Blind super-resolution	Framework description
ScUnicorn	TBD	Yes	**Yes** ^1^	CNN-based method
Capricorn (Fang *et al.* 2024)	Jun. 2024	No	No	Diffusion-based method
HiCDiff (Wang and Cheng 2024)	Jun. 2024	Yes	No	Diffusion-based method
ScHiCEDRN (Wang *et al.* 2023)	Jul. 2023	Yes	No	Residual GAN-based method
HiCARN (Hicks and Oluwadare 2022)	Mar. 2022	No	No	CNN & GAN-based method
EnHiC (Hu and Ma 2021)	Jul. 2021	No	No	GAN-based method
VEHiCLE (Highsmith and Cheng 2021)	Apr. 2021	No	No	GAN-based method
HiCSR (Dimmick 2020)	Jul. 2020	No	No	GAN-based method
SRHiC (Li and Dai 2020)	Apr. 2020	No	No	CNN-based method
DeepHiC (Hong *et al.* 2020)	Feb. 2020	No	No	GAN-based method method
HiCNN2 (Liu and Wang 2019b)	Oct. 2019	No	No	CNN-based method
HiCNN (Liu and Wang 2019a)	Apr. 2019	No	No	CNN-based method method
HiCGAN (Liu *et al.* 2019)	Jul. 2019	No	No	GAN-based method
Loopenhance (Zhang *et al.* 2022)	Jul. 2018	Yes	No	CNN-based method
HiCPlus (Zhang *et al.* 2018b)	Feb. 2018	No	No	CNN-based method.

1 Bold text for emphasis.

## 2 Materials and methods

The analysis of single-cell Hi-C data requires innovative methods to address challenges such as noise, sparsity, and the lack of high-resolution interaction maps. Our proposed algorithm, Unicorn, offers a two-in-one solution for scHi-C data analysis: (i) ScUnicorn, which enhances Hi-C data through blind super-resolution, and (ii) 3DUnicorn, which reconstructs 3D chromosome structures using a Maximum Likelihood Algorithm (Oluwadare *et al.* 2018) from the enhanced scHi-C data. Together, these methods deliver accurate, high-quality insights into chromosomal interactions and spatial organization.

### 2.1 ScUnicorn: blind super-resolution algorithm for scHi-C data enhancement

Super-resolution algorithms aim to reconstruct high-resolution (HR) images or matrices from their low-resolution (LR) counterparts. In the context of scHi-C data, super-resolution holds immense promise for overcoming the challenges of sparse and noisy interaction maps (Liu and Wang 2019a, Hong *et al.* 2020, Li and Dai 2020). However, early super-resolution techniques, particularly in computer vision, primarily relied on supervised learning methods, which depend heavily on the availability of pre-defined pairs of LR-HR datasets (Zhang *et al.* 2018b, Luo *et al.* 2020). Blind super-resolution emerged as a solution to this challenge, introducing methods that estimate both the HR data and the underlying degradation function simultaneously (Shocher *et al.* 2018, Zhang *et al.* 2018a). This paradigm shift allowed super-resolution techniques to generalize better to real-world data. In genomic research, it is also less feasible to have pre-defined pairs of LR-HR datasets, making blind super-resolution the most practical approach for efficient scHi-C data enhancement.

**Figure 1.**
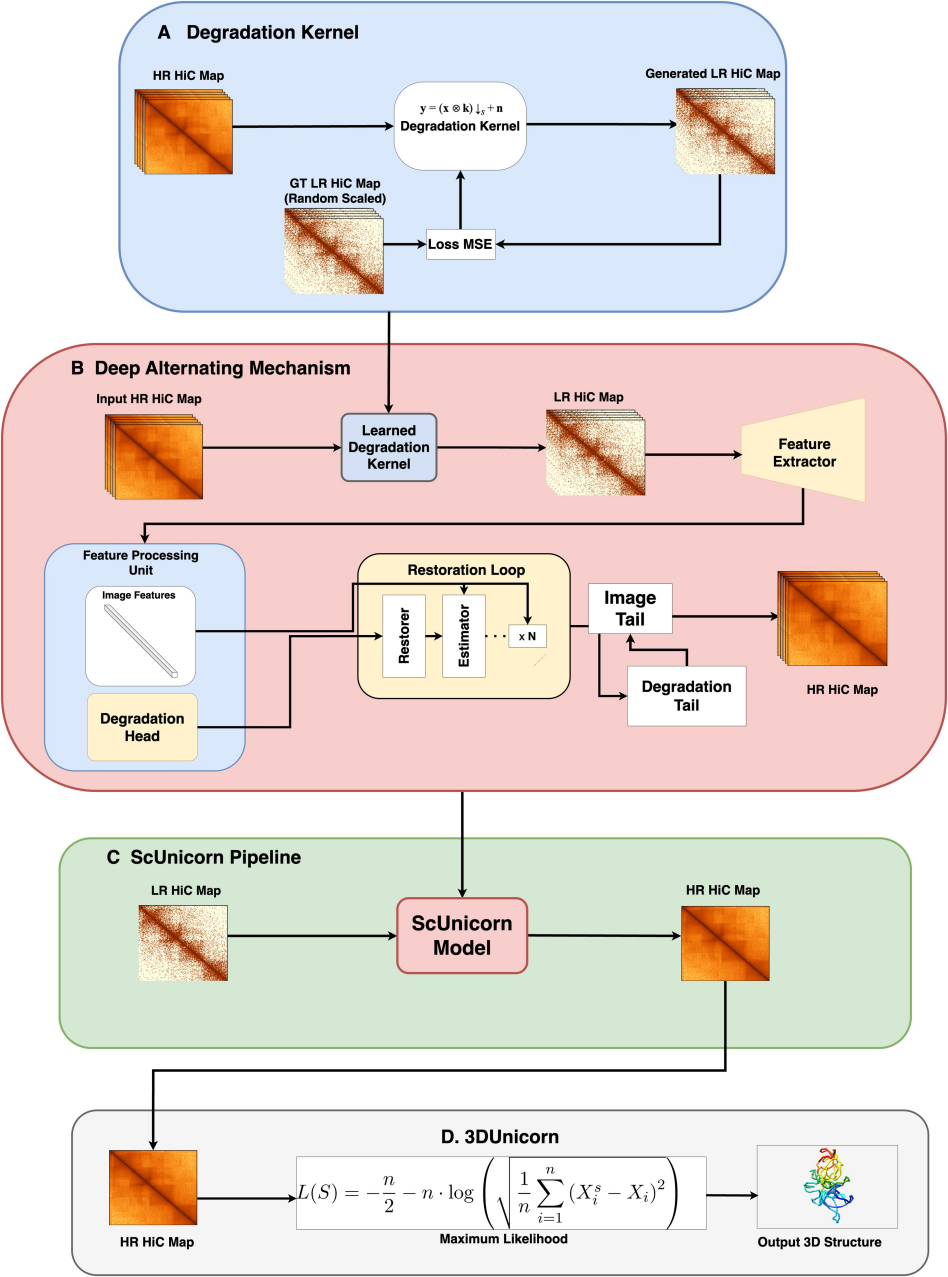
Unicorn pipeline consisting of ScUnicorn and 3DUnicorn algorithms. (A) Degradation Kernel: shows the degradation kernel learning process where it takes HR Hi-C maps and learns the optimal kernel to downscale it to a degraded LR Hi-C map. (B) Deep Alternating Mechanism: includes feature processing, restoration loops with iterative estimation and recovery, and final output refinement to enhance the resolution of Hi-C data. (C) ScUnicorn Pipeline: encapsulates the entire process where we input an LR Hi-C map to the trained ScUnicorn model and get an enhanced scHi-C map. (D) The 3DUnicorn module utilizes the enhanced scHi-C map for 3D chromosome structure generation, using a Maximum Likelihood algorithm to accurately infer 3D spatial conformations of chromatin (Oluwadare *et al.* 2018). This updated framework highlights modular design, dynamic adaptation to input data, and robust resolution enhancement while preserving critical biological insights.

In this study, we propose ScUnicorn, a novel algorithm for scHi-C data enhancement based on a blind super-resolution approach (Fig. 1A–C). Specifically, ScUnicorn draws inspiration from the Deep Alternating Network (DAN) (Luo *et al.* 2020), which addresses challenges in super-resolution by jointly optimizing kernel estimation and image restoration. Traditional methods separate these steps, leading to potential incompatibility and error propagation. Luo *et al.* (2020) resolves this by alternating between two modules: an *Estimator* that predicts the blur kernel and a *Restorer* that reconstructs the super-resolution image using the estimated kernel. This iterative process ensures better compatibility and robustness in the model. ScUnicorn, an algorithm built on these principles, is specifically adapted to handle the unique characteristics of scHi-C data. It provides a robust framework capable of dynamically adjusting to the input matrices, enabling the reconstruction of high-resolution genomic data that preserves essential biological insights while addressing issues like noise and sparsity. Through this approach, ScUnicorn enhances scHi-C data while maintaining biological fidelity, making it an ideal tool for advancing 3D genome structure analysis.

Also, traditional methods for Hi-C enhancement often use degradation algorithms with static parameters, e.g. 1/16 or 1/32, which limits their ability to adapt to varying input data (Wang *et al.* 2023, Wang and Cheng 2024). In contrast, ScUnicorn uses a dynamic and learnable degradation model that optimizes the transformation process during training. ScUnicorn optimizes its degradation kernel and super-resolution reconstruction in an end-to-end trainable framework. Paired LR and HR training data are used to simulate realistic LR-HR transformations, allowing ScUnicorn to adapt dynamically and achieve superior reconstruction accuracy. Unlike methods relying on predefined degradation models (Table 1), ScUnicorn integrates information from both LR and estimated HR matrices during training, enhancing kernel estimation and resolution restoration. This flexibility ensures accurate and consistent super-resolution outcomes (Luo *et al.* 2020), making ScUnicorn ideal for a range of Hi-C datasets, including those with sparse or unavailable high-quality HR data (Beagrie *et al.* 2017, Stevens *et al.* 2017).

#### 2.1.1 ScUnicorn architecture

The ScUnicorn model introduces a novel approach to Hi-C super-resolution by dynamically learning and optimizing the degradation kernel, ensuring robust resolution enhancement tailored to the unique characteristics of Hi-C data. This design overcomes limitations of existing methods, such as fixed-scale downsampling, by introducing a flexible framework where the optimal degradation scale is learned directly from the data.

**(A) Dynamic degradation kernel learning**

A central innovation of ScUnicorn is its dynamic degradation kernel as seen in Fig. 1A. Unlike traditional methods that rely on fixed downsampling scales (e.g. 1/16 or 1/32), ScUnicorn learns the degradation kernel in an end-to-end trainable framework. HR Hi-C input contact map is paired with randomly downscaled LR data as ground truth (GT) before the training process begins. The degradation kernel is trained to transform the HR input into a degraded, generated LR Hi-C map version that matches the randomly downscaled GT LR data. In Equation (1), the ScUnicorn model jointly estimates the HR Hi-C matrix, denoted as H, and the degradation kernel, denoted as T. The observed LR Hi-C matrix, denoted as L is modeled as:


(1) L=T(H)+E,

where E accounts for noise or distortions. The objective function, equation (2), balances reconstruction fidelity and regularization:


(2) $$J(H,T)=∥L-T(H){∥}^{2}+\beta P(H),$$

where $∥L-T(H){∥}^{2}$ ensures consistency between the simulated LR data and the observed input, P(H) imposes constraints such as sparsity or smoothness, and β controls the regularization strength.

**(B) Deep alternating mechanism**

The architecture of ScUnicorn, as illustrated in Fig. 1B, comprises several key components designed to iteratively refine both the degradation kernel and the super-resolved Hi-C map. Initially, the HR Hi-C map is transformed into a LR (degraded), Hi-C map using the Learned Degradation Kernel from Step A. The features are then passed on to be processed iteratively across several modules.

- *Degradation head:* Uses features from the LR Hi-C map produced by the degradation kernel. These features are then processed and refined to capture critical interaction patterns necessary for restoration (Zhang *et al.* 2017).
- *Restoration loop:* Includes two core modules:
  - *Estimator:* Dynamically estimates and refines the features from the LR Hi-C map, ensuring iterative improvement (Luo *et al.* 2020). Equation (3) shows the steps involved:
    (3) $$T^{\left(k+1\right)}=\underset{T}{\mathrm{arg min}}∥L-T(H^{\left(k+1\right)}){∥}^{2},$$
    where L is the LR Hi-C map, H is the HR map, and T is the degradation kernel.
  - *Restorer:* Progressively reconstructs high-frequency details of the Hi-C map, enhancing its resolution using the refined degradation kernel (Zhang *et al.* 2018a). Equation (4) shows the steps involved:
    (4) $$H^{\left(k+1\right)}=\underset{H}{\mathrm{arg min}}∥L-T^{\left(k\right)}(H){∥}^{2}+\beta P(H),$$

where P(H) imposes constraints, such as sparsity or smoothness, on the reconstructed HR map.

This alternating optimization between the *Estimator* and *Restorer* continues iteratively and is repeated *N* times. In this work we used N=5 based on an extensive ablation study reported by Luo *et al.* (2020), allowing ScUnicorn to dynamically refine both the HR Hi-C map and the degradation kernel until convergence.

- *Degradation tail:* Validates the reconstructed HR map by ensuring consistency with the observed LR data, reducing overfitting to the degradation process (Zhang *et al.* 2017).
- *Image tail:* Finalizes the reconstructed HR Hi-C map to ensure it meets biological fidelity standards while suppressing noise (Huang *et al.* 2020).

Ultimately, the architecture and workflow of ScUnicorn, including the dynamic degradation kernel and restoration loop, are depicted in Fig. 1A and B. Finally, Fig. 1C shows how the trained model using the ScUnicorn Steps A and B is used for scHi-C data enhancement.

### 2.2 3DUnicorn: maximum likelihood algorithm for single cell Hi-C data 3D chromosome structure reconstruction

Hi-C experiments provide genome-wide insights into interactions between chromosome regions, which are crucial for understanding spatial organization and its role in biological processes like gene regulation, genome folding, and function. As previously mentioned, one of the main challenges in modeling 3D chromosome structures is addressing data sparsity. With ScHi-C data now enhanced by ScUnicorn, this issue has been tackled, but the next challenge is translating these interaction frequencies into precise spatial distances to reconstruct accurate 3D models. To achieve this, we adopt the maximum likelihood algorithm by Oluwadare *et al.* (2018), designed to reconstruct chromosome 3D structures using chromosomal contact data. ScUnicorn uses the iterative maximum likelihood method to convert interaction frequencies into spatial distances, automatically estimating the conversion factor, α, which governs the relationship between these frequencies and the resulting distances.

#### 2.2.1 Log-likelihood objective function

The 3DUnicorn algorithm relies on a log-likelihood objective function that works to align a predicted 3D structure with the contact matrix data (Oluwadare *et al.* 2018). In this method, each entry in the contact matrix is viewed as a distance between chromosomal regions. The primary goal is to reduce the differences between the model-generated spatial distances and the observed interaction frequencies from the matrix following equation (5).


(5) $$L(S)=-\frac{n}{2}-n\cdot \;\text{log}\;\left(\sqrt{\frac{1}{n}\sum_{i=1}^{n}{\left(X_{i}^{s}-X_{i}\right)}^{2}}\right)$$

Here, $X_{i}^{s}$ represents the spatial distance from 3D coordinates, $X_{i}$ denotes the wish distance from Hi-C data, and *n* is the number of distance pairs used in the loss computation. The algorithm refines the 3D structure using gradient descent until convergence.

### 2.3 Metrics for ScUnicorn HR Hi-C map evaluation

Two widely used metrics for evaluating the quality of HR images are Peak Signal-to-Noise Ratio (PSNR) and Structural Similarity Index Measure (SSIM):

#### 2.3.1 Peak signal-to-noise ratio

PSNR measures the ratio between a signal’s maximum possible power and noise’s power that affects its representation. It is expressed in decibels (dB). A higher PSNR value indicates that the HR image is closer to the ground truth. For Hi-C maps, PSNR helps evaluate how effectively the model reduces noise and restores missing data in LR inputs. The formula for PSNR is given by Equation (6):


(6) $$\text{PSNR}=10\cdot {\;\text{log}\;}_{10}\left(\frac{{\text{MAX}}^{2}}{\text{MSE}}\right),$$

where:

- MAX is the maximum possible pixel value of the image (e.g. 1 for normalized data or 255 for 8-bit images).
- MSE is the mean squared error between the super-resolved image $I_{\text{SR}}$ and the ground truth $I_{\text{GT}}$, defined as:
  (7) $$\text{MSE}=\frac{1}{N}\sum_{i=1}^{N}{\left(I_{\text{SR},i}-I_{\text{GT},i}\right)}^{2},$$

where *N* is the total number of pixels.

#### 2.3.2 Structural similarity index measure

The SSIM is a metric that quantifies the similarity between a reconstructed high-resolution (HR) image and its reference by analyzing luminance, contrast, and structural components. Unlike conventional metrics such as PSNR, SSIM prioritizes the preservation of spatial relationships and patterns, which are essential for capturing biologically meaningful interactions in Hi-C data. A higher SSIM value reflects better retention of the underlying structural features in the enhanced interaction maps. The mathematical definition of SSIM is given by Equation (8):


(8) $$\text{SSIM}(p,q)=\frac{\left(2\bar{p}\bar{q}+D_{1})(2\delta_{pq}+D_{2}\right)}{\left({\bar{p}}^{2}+{\bar{q}}^{2}+D_{1})(\delta_{p}^{2}+\delta_{q}^{2}+D_{2}\right)},$$

where:

-
$\bar{p}$ and $\bar{q}$: Mean intensity values of the matrices *p* and *q*.
-
$\delta_{p}^{2}$ and $\delta_{q}^{2}$: Variance of the matrices *p* and *q*.
-
$\delta_{pq}$ : Covariance between *p* and *q*.
-
$D_{1}={\left(\alpha \cdot M\right)}^{2}$ and $D_{2}={\left(\beta \cdot M\right)}^{2}$: Stabilization constants that prevent division by zero, where *M* represents the range of matrix values (e.g. 1 or 255), and α,β are small constants (e.g. α=0.01,β=0.03).

#### 2.3.3 GenomeDISCO

GenomeDISCO (Ursu *et al.* 2018) is a specialized evaluation metric designed to assess the reproducibility and biological consistency of Hi-C contact maps, particularly for datasets with high levels of sparsity and noise. It works by comparing interaction patterns between two Hi-C maps through iterative smoothing, which emphasizes meaningful chromatin interactions while minimizing the effects of random noise. For ScUnicorn, GenomeDISCO is vital as it measures how well the super-resolution process retains critical biological structures in the enhanced Hi-C maps.

### 2.4 Metric for 3DUnicorn chromosome 3D reconstruction evaluation

The metric used to evaluate the quality of reconstructed chromosome 3D structures is Pearson’s Correlation Coefficient:

#### 2.4.1 Pearson correlation coefficient

Pearson correlation coefficient (PCC) evaluates the linear relationship between two variables, specifically the reconstructed distances from the 3D structure and the target distances derived from Hi-C contact frequencies. It provides insights into how well the 3D model aligns with biological data. This metric is critical for evaluating the accuracy of 3D chromosomal reconstructions, with higher values reflecting stronger alignment between the spatial model and Hi-C interaction data. The formula for Pearson correlation is given as equation 9:


(9) $$r=\frac{\sum_{i=1}^{n}\left(X_{i}-\bar{X}\right)(Y_{i}-\bar{Y})}{\sqrt{\sum_{i=1}^{n}{\left(X_{i}-\bar{X}\right)}^{2}\sum_{i=1}^{n}{\left(Y_{i}-\bar{Y}\right)}^{2}}},$$

where:

- *r*: Pearson correlation coefficient.
-
$X_{i}$ : Reconstructed distances (e.g. computed from the 3D structure).
-
$Y_{i}$ : Target distances (e.g. derived from Hi-C contact frequencies).
-
$\bar{X}$ : Mean of reconstructed distances (*X*).
-
$\bar{Y}$ : Mean of target distances (*Y*).
- *n*: Number of data points (e.g. pairwise loci comparisons).

## 3 Results

### 3.1 Dataset overview

In this study, we used the scHi-C dataset of human frontal cortex (GSE130711) (Lee *et al.* 2019), and Drosophila melanogaster (Ulianov *et al.* 2021). Additionally, we used the mouse embryonic stem cells (mESCs) cell 1, chromosome 3 and 11 datasets from Stevens *et al.* (2017). Chromosomes 3 and 11 were selected because 3D-FISH validation data is available only for these chromosomes (Beagrie *et al.* 2017), making them the only viable options for independent validation of 3D chromosome reconstruction outputs. Additionally, their diverse chromatin interaction profiles make them well-suited for benchmarking 3D genome reconstruction methods.

### 3.2 ScUnicorn outperforms state-of-the-art single-cell enhancement methods

For this analysis, we benchmarked the performance of ScUnicorn against other state-of-the-art single-cell Hi-C enhancement algorithms: Loopenhance (Zhang *et al.* 2022), ScHiCEDRN (Wang *et al.* 2023), and HiCDiff (Wang and Cheng 2024). These were selected based on their applicability to single-cell Hi-C data enhancement. Unlike bulk Hi-C methods, these models are designed specifically to handle the sparsity and noise in scHi-C data.

**Figure 2.**
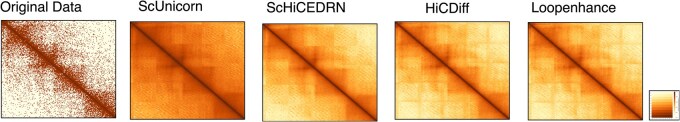
Comparison of Hi-C matrix heatmaps for different methods. From left to right: Original Data, ScUnicorn (proposed method), ScHiCEDRN, HiCDiff, and Loopenhance for Mouse Data chr 11 at 100 KB. The color bar on the right indicates the contact frequency (log10 scale) .

All algorithms, including ScUnicorn, were trained on human frontal cortex chromosome data (chromosomes 1, 3, 5, 7, 8, 9, 11, 13, 15, 16, 17, 19, 21, and 22), with other methods using downsampling to 0.75. ScUnicorn, however, applies a dynamic degradation approach rather than predefined downsampling, leading to more flexible adaptation to varying data characteristics. We performed blind validation on mESC chromosome 11 at 500 KB and 100 KB resolutions to assess cross-species generalization (Table 2). ScUnicorn outperforms all other methods in PSNR, SSIM, and GenomeDisco scores, demonstrating its superior ability to enhance single-cell Hi-C resolution. Given that scUnicorn has demonstrated robust performance at 100 KB and 500 KB resolutions, we expect that this trend will continue at higher resolutions, where the increased signal-to-noise ratio can further enhance structural consistency and model reliability (Banecki *et al.* 2024). Figure 2 shows the heatmap visualization generated by different methods for a randomly selected 40 x 40 regions for chromosome 11 at 100 KB. Similarly, in another cross-species evaluation, ScUnicorn excelled on Drosophila chromosome 2L using human frontal cortex chromosome training data, outperforming methods like ScHiCEDRN, Loopenhance, and HiCDiff, as detailed in Table 3. These results underscore the effectiveness of the degradation module and kernel optimizations integrated into ScUnicorn.

**Table 2.** Evaluation of single-cell super-resolution methods on mESC Chromosome 11 scHi-C data at 500 KB and 100 KB resolutions.^a^

500 KB resolution			
Model	PSNR	SSIM	GenomeDISCO
Loopenhance	26.82	0.828	0.808
ScHiCEDRN	27.94	0.855	0.825
ScUnicorn	**30.12** ^b^	**0.901** ^b^	**0.885** ^b^
HiCDiff	28.90	0.878	0.850

100 KB resolution			
Model	PSNR	SSIM	GenomeDISCO
Loopenhance	26.15	0.820	0.805
ScHiCEDRN	27.45	0.850	0.822
ScUnicorn	**29.50** ^b^	**0.895** ^b^	**0.885** ^b^
HiCDiff	27.80	0.870	0.840

a ScUnicorn consistently achieves the highest scores across both resolutions, demonstrating its ability to produce high-quality Hi-C data while preserving biological insights.

b Emphasis for highest score.

**Table 3.** Evaluation of single-cell super-resolution methods on Drosophila Melanoglaster Chromosome 2L scHi-C data.

Model	PSNR	SSIM	GenomeDISCO
Loopenhance	29.50	0.800	0.800
ScHiCEDRN	30.50	0.815	0.808
ScUnicorn	**31.23** ^a^	**0.897** ^a^	**0.830** ^a^
HiCDiff	28.80	0.860	0.825

a Emphasis on highest score.

Topologically Associating Domains (TADs) play a critical role in chromatin organization, influencing gene regulation and structural integrity in Hi-C data. A key aspect of model performance lies in preserving these domains, which serve as biologically meaningful structures. To validate the effectiveness of ScUnicorn, we performed a TAD analysis, using DeDoc2 (Li *et al.* 2023), a graph-based TAD detection algorithm for TAD detection from scHi-C datasets, on the predicted contact matrices, comparing them with ScHiCEDRN, Loopenhance, and HiCDiff (Supplementary Fig. S1). We quantified the consistency of structural preservation with the original scHi-C matrix by calculating the L2 norm of insulation scores, which reflects the stability of TAD boundaries. Lower L2 norm values indicate greater consistency in preserving chromatin topology. As shown in Supplementary Fig. S2, ScUnicorn achieves the lowest L2 norm for both chromosome 3 and chromosome 11, demonstrating its superior ability to maintain topological structures. These results confirm that ScUnicorn not only enhances resolution but also preserves key chromatin features, unlike other methods that may introduce artifacts or disrupt structural integrity. To further validate the performance of ScUnicorn beyond single-cell Hi-C data, we evaluated its effectiveness on bulk Hi-C data (Supplementary Section S1). The results, summarized in Supplementary Table S1, demonstrates that ScUnicorn generalizes effectively to bulk Hi-C applications, although designed for single-cell Hi-C data, providing robust and reliable performance across different data types.

### 3.3 3DUnicorn: reconstruction and validation of chromatin structures with 3D FISH data

The 3DUnicorn model reconstructs 3D chromatin structures from scHi-C data. In this study, we used the enhanced ScUnicorn contact matrices as input for the maximum likelihood algorithm to create 3D structures of mESC scHi-C data chromosomes 3 and 11 from Stevens *et al.* (2017) at 500 KB and 100 KB resolutions. Thereafter, the output 3D structures results were validated using experimental 3D FISH data (Beagrie *et al.* 2017). In this work, we benchmarked our method against SCL (Zhu and Wang 2019), which is recognized as one of the state-of-the-art algorithms for single-cell genome structure reconstruction due to its effective handling of sparse scHi-C data and its demonstrated accuracy in 3D genome modeling.

To assess the consistency of our reconstructed chromatin models and account for the inherent molecular variability in chromatin structure, we generated an ensemble of 20 structural models for each chromosome. We used the TM-score (Zhang and Skolnick 2004) to quantify the structural similarity between each model and all other models in the ensemble. The TM-score is a widely used metric for assessing the similarity between two protein 3D structures, where higher values (closer to 1) indicate greater similarity and structural consistency. For each model, we calculated its average TM-score against all other models in the ensemble as a measure of reproducibility and consistency of our algorithm. The results for chromosomes 3 and 11, comparing 3DUnicorn and SCL, are provided in the supplementary document (Supplementary Tables S2 and S3, respectively). Our findings demonstrate high reproducibility for 3DUnicorn, with the lowest pairwise TM-scores among the 20 structures being 0.986 for chromosome 3 and 0.975 for chromosome 11. In comparison, SCL recorded lowest TM-scores of 0.973 for chromosome 3 and 0.969 for chromosome 11. These consistently high minimum scores indicate that 3DUnicorn reliably produces structurally similar models across different runs, despite potential molecular variability, highlighting the robustness and stability of the reconstruction process. Additionally, we present a box plot showing the pairwise TM-score comparisons of each structural model against all other models in the ensemble for chromosome 3 and 11 (Supplementary Fig. S3). The consistently high TM-scores shown in the box plot further indicate structural agreement across models, despite some molecular variability. These results highlight the robustness and stability of 3DUnicorn in generating reproducible chromatin structures.

**Figure 3.**
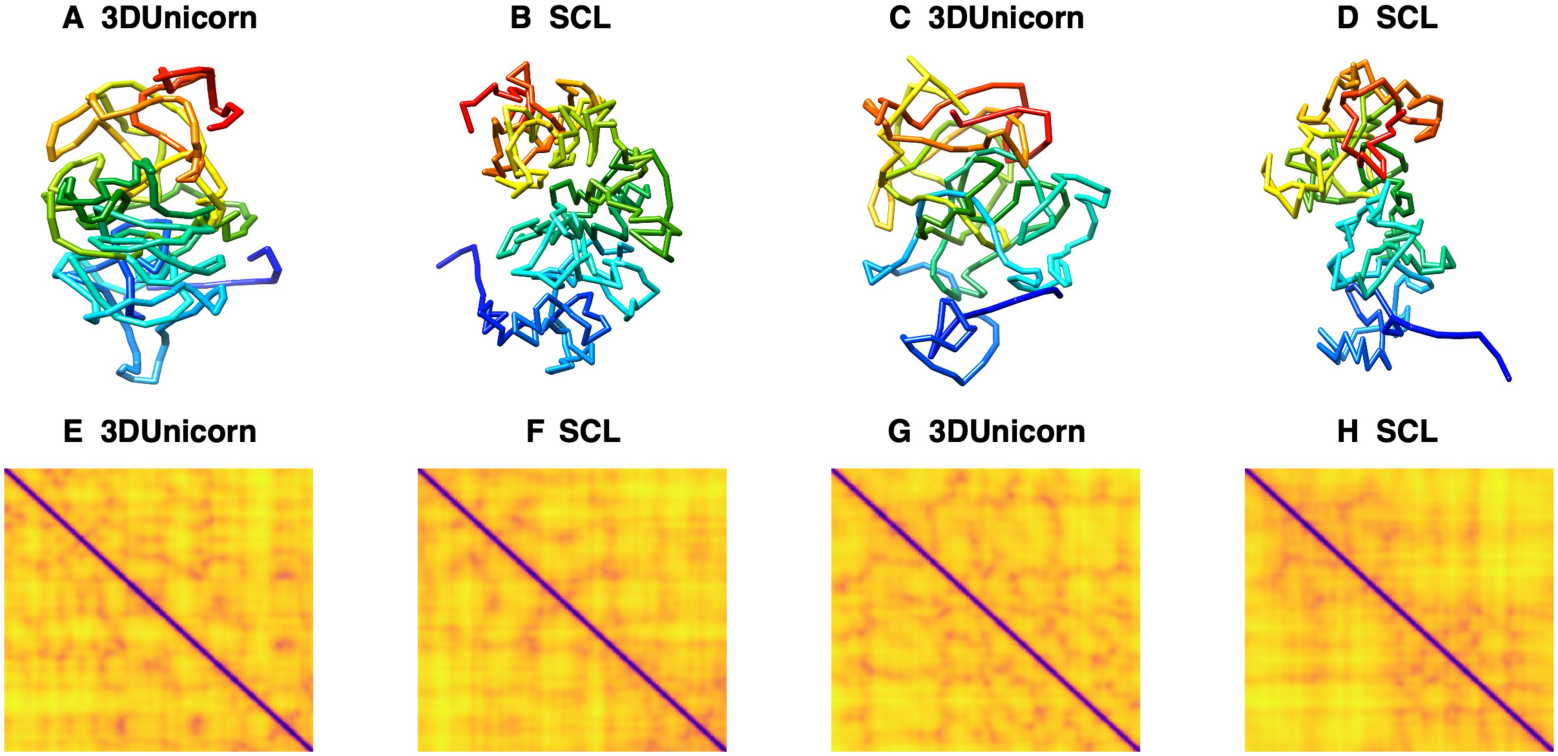
Comparison of 3D structures and their corresponding spatial distance heatmaps reconstructed using 3DUnicorn and SCL for Chromosome 3 and Chromosome 11 at 500 KB resolution. Panels (A) and (C) show the 3DUnicorn-reconstructed 3D structures for Chromosomes 3 and 11, while (B) and (D) present the corresponding structures from SCL. Heatmaps for Chromosomes 3 and 11, generated using 3DUnicorn, are displayed in (E) and (G), and those from SCL are shown in (F) and (H).

Tables 4 and 5 provide a summary of the distances between the probe pairs for both chromosomes at resolutions of 500 KB and 100 KB, based on one representative model. The distances reconstructed using 3DUnicorn were validated against experimental FISH (Beagrie *et al.* 2017) data by calculating the PCC between the reconstructed distances and the measured values from 3D FISH experiment. For Chromosome 3, 3DUnicorn achieved a PCC of 0.987 (Fig. 3A), outperforming SCL, which achieved a PCC of 0.803 (Fig. 3B). For Chromosome 11, 3DUnicorn achieved a PCC of −0.133 (Fig. 3C), which, although negative, still surpassed PCC of SCL −0.729 (Fig. 3D at 500 KB resolution). At 100 KB resolution, for Chromosome 3, 3DUnicorn achieved a PCC of 0.809, compared to the PCC of SCL of 0.559. Similarly, for Chromosome 11, 3DUnicorn achieved a PCC of 0.867, marginally outperforming SCL, which achieved a PCC of 0.860.

**Table 4.** Distances between two fluorescence in situ hybridization (FISH) probes in the models at a 100 KB resolution for Chromosomes 3 and 11.^a^

Chromosome	L1 (Position, MB)	L2 (Position, MB)	3DUnicorn (L1-L2)	SCL (L1-L2)	FISH (L1-L2)
**3**	87.93–88.43	91.07–91.57	0.1	0	2.6
	87.93–88.43	102.58–103.08	0.2	0.1	14.1
	87.93–88.43	96.32–96.82	0.2	0	7.9
	96.32–96.82	102.58–103.08	0.1	0.1	5.8
**11**	52.15–52.65	41.4–41.9	0.3	0.2	10.2
	33.28–33.78	41.4–41.9	0.3	0.1	7.6
	33.28–33.78	52.15–52.65	0.3	0.3	18.4
	33.28–33.78	61.88–62.38	0.4	0.3	28.1

a The table presents the genomic positions of two probes (L1 and L2) located on selected loops of Chromosomes 3 and 11. Columns 2–3 display the start and end positions of each probe in megabases (MB). Columns 4–6 provide the distances between the two probes as calculated using 3DUnicorn, SCL (Zhu and Wang 2019), and FISH experiments (Beagrie *et al.* 2017).

**Table 5.** Distances between two fluorescence in situ hybridization (FISH) probes in the models at a 500 KB resolution for Chromosomes 3 and 11.^a^

Chromosome	L1 (Position, MB)	L2 (Position, MB)	3DUnicorn (L1-L2)	SCL (L1-L2)	FISH (L1-L2)
**3**	87.93–88.43	91.07–91.57	1.4	0.3	2.6
	87.93–88.43	102.58–103.08	2.2	0.5	14.1
	87.93–88.43	96.32–96.82	1.7	0.4	7.9
	96.32–96.82	102.58–103.08	1.5	0.2	5.8
**11**	52.15–52.65	41.4–41.9	3.1	0.7	10.2
	33.28–33.78	41.4–41.9	2.0	0.6	7.6
	33.28–33.78	52.15–52.65	1.8	0.3	18.4
	33.28–33.78	61.88–62.38	2.4	0.4	28.1

a The table presents the genomic positions of two probes (L1 and L2) located on selected loops of Chromosomes 3 and 11. Columns 2–3 display the start and end positions of each probe in megabases (MB). Columns 4–6 provide the distances between the two probes as calculated using 3DUnicorn, SCL (Zhu and Wang 2019), and FISH experiments (Beagrie *et al.* 2017).

These results highlight the strong alignment between computational models from 3DUnicorn and biological data compared to existing state-of-the-art algorithm. At 100 KB resolution, the reconstructed distances showed closer alignment with experimental FISH data, underscoring the value of higher-resolution models to accurately capture detailed chromatin architecture. At 500 KB resolution, Chromosome 3 performed exceptionally well, achieving a strong Pearson’s correlation. These findings confirm the effectiveness of 3DUnicorn in creating biologically accurate 3D chromatin structures, making it a valuable tool for single-cell genomic studies. Figure 3 visually illustrates the reconstructed 3D structures.

## 4 Conclusion

In this study, we propose an algorithm, Unicorn, which combines the ScUnicorn and 3DUnicorn algorithms for single-cell Hi-C data analysis. ScUnicorn demonstrates that incorporating a high-performance degradation kernel capable of learning to generate optimally degraded Hi-C contact maps enables our alternating network to produce high-resolution contact matrices. This approach effectively eliminates the reliance on predefined downsampling scales. Furthermore, the proposed model consistently outperforms state-of-the-art supervised super-resolution networks in scHi-C contact matrix imputation tasks, thereby validating its superior performance. The dynamic degradation kernel represents a significant advancement in Hi-C super-resolution. By learning an optimal degradation process tailored to each dataset, ScUnicorn surpasses the limitations of fixed-scale downsampling, achieving superior reconstruction performance. This innovation ensures that the reconstructed HR Hi-C maps retain critical biological insights while reducing noise and sparsity, making ScUnicorn uniquely suited for diverse Hi-C datasets. Also, we validated the results of 3DUnicorn by comparing the distances derived from our method with experimental 3D-FISH data (Beagrie *et al.* 2017) for chromosomes 3 and 11. Our results show a strong alignment between the reconstructed distances and experimental values. In conclusion, the accuracy of the results obtained from the 3DUnicorn algorithm on the HR Hi-C maps from ScUnicorn further validates Unicorn’s performance in capturing chromatin spatial organization across varying resolutions and its applicability for downstream analyses.

## Supplementary Material

btaf177_Supplementary_Data

## Data Availability

The Unicorn implementation source code for both the ScUnicorn and 3DUnicorn algorithms is publicly accessible at: https://github.com/OluwadareLab/Unicorn. The datasets used in this study for training, validation and testing is available here at this Zenodo link: https://doi.org/10.5281/zenodo.14750809. Also, we used preprocessed Drosophila melanogaster data from Ulianov *et al.* (2021), as provided by Wang *et al.* (2023), available at: https://doi.org/10.5281/zenodo.10535486.
