# Supplementary material for: Unicorn: enhancing single-cell Hi-C data with blind super-resolution for 3D genome structure reconstruction
Source: Bioinformatics. 2025 Jul 15;41(Suppl 1):i475–83. doi: 10.1093/bioinformatics/btaf177 (PMC12261411; doi:10.1093/bioinformatics/btaf177)
Supplement: btaf177_Supplementary_Data [file btaf177_supplementary_data.zip › Oluwadare.344.sup.1.pdf]

SUPPLEMENTARY MATERIAL

Unicorn: Enhancing Single-Cell Hi-C Data with Blind Super-Resolution for 3D Genome Structure Reconstruction

Mohan Kumar B Chandrashekar,[1,†] Rohit Menon,[1,†] Samuel Olowofila[1] and Oluwatosin Oluwadare[1,2,*]

[1]Department of Computer Science, University of Colorado at Colorado Springs, 1420 Austin Bluffs Pkwy, Colorado Springs, 80918, Colorado, USA and [2]Department of Biomedical Informatics, University of Colorado Anschutz Medical Campus, 13001 East 17th Place, Aurora, 80045, Colorado, USA

*Corresponding author. ooluwada@uccs.edu. † These authors contributed equally to this work.

1. Evaluating ScUnicorn's Generalization to Bulk Hi-C Dataset

To further validate the performance of ScUnicorn beyond single-cell Hi-C data, we evaluated its effectiveness on bulk Hi-C data. We trained all models on GM12878 human lymphoblastoid cell line data (GSE63525) [4], using chromosomes 2, 3, 4, 5, 6, 7, 8, 9, 10, and 11, and tested on chromosome 12. The models compared include HiCARN [1], DeepHiC [2], and ScUnicorn. The input data was downsampled by a factor for 0.75 for HiCARN and DeepHiC. The results, summarized in Table 1, demonstrate that ScUnicorn achieves performance close to HiCARN, the state-of-the-art bulk Hi-C enhancement model, while outperforming DeepHiC across all metrics. This suggests that, although designed for single-cell Hi-C data, ScUnicorn generalizes effectively to bulk Hi-C applications, providing robust and reliable performance across different data types.

Table 1. Performance comparison of ScUnicorn, HiCARN, and DeepHiC on Bulk Hi-C data GM12878 chromosome 12. It demonstrates ScUnicorn's ability to generalize beyond single-cell data (* indicates a method not originally designed for bulk Hi-C data enhancement).

Model	PSNR	SSIM	GenomeDisco
HiCARN	**30.5**	**0.890**	**0.845**
DeepHiC	26.7	0.810	0.780
ScUnicorn*	29.8	0.875	0.830

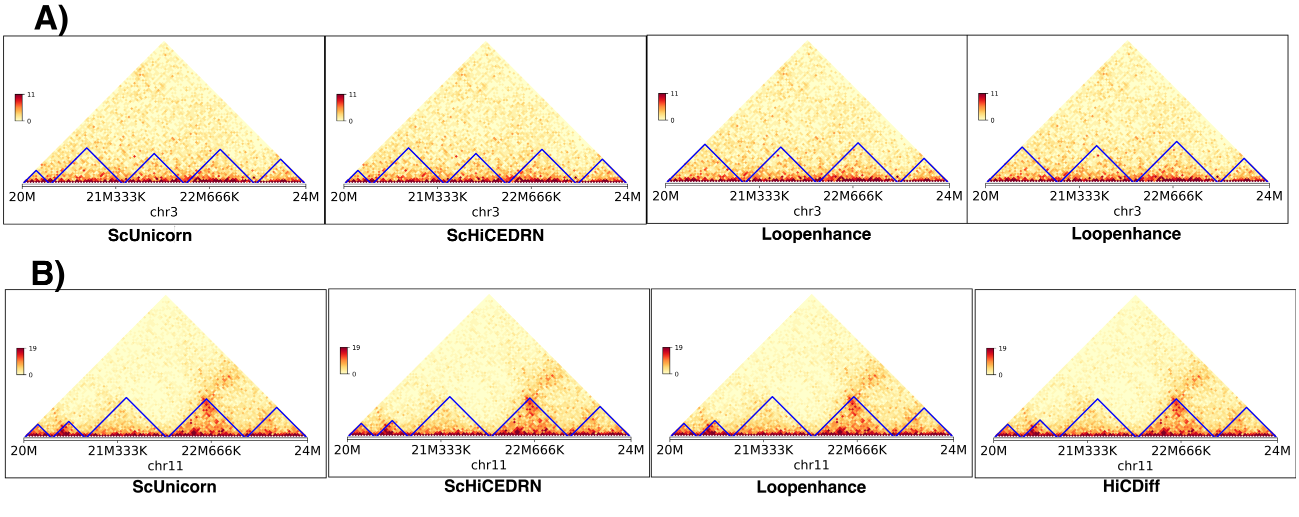

Fig. 1. Topologically Associating Domains (TADs) in Predicted Hi-C Matrices. The figure presents TAD predictions across different models for mouse chromosome 3 (A) and chromosome 11 (B), generated using the DeDoc2 algorithm [3] The models compared include ScUnicorn, ScHiCEDRN, Loopenhance, and HiCDiff

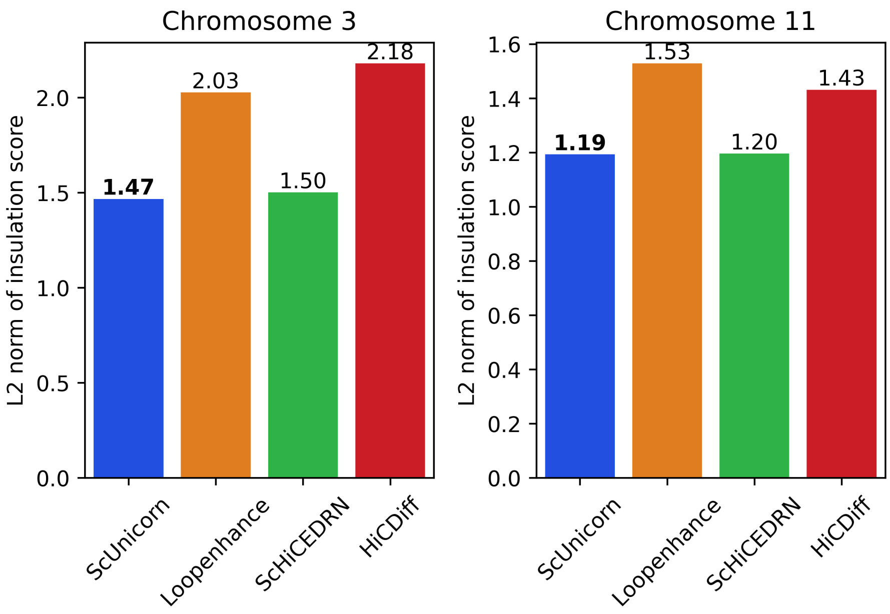

Fig. 2. The L2 norm plot of TAD boundaries insulation score for mESc chromosome 3 and 11 The bar plots show the L2 norm of insulation scores for different models on mouse chromosome 3 (left) and chromosome 11 (right). The models compared include ScUnicorn, Loopenhance, ScHiCEDRN, and HiCDiff. A lower L2 norm suggests better TAD boundary preservation, indicating superior structural consistency.

3DUnicorn PDB	TMScore average
chr3_500kb_7.pdb	0.991425
chr3_500kb_16.pdb	0.990460
chr3_500kb_9.pdb	0.991243
chr3_500kb_19.pdb	0.990789
chr3_500kb_3.pdb	0.990391
chr3_500kb_1.pdb	0.988604
chr3_500kb_13.pdb	0.989385
chr3_500kb_12.pdb	0.989212
chr3_500kb_11.pdb	0.987794
chr3_500kb_5.pdb	0.987621
chr3_500kb_17.pdb	0.988234
chr3_500kb_15.pdb	0.987404
chr3_500kb_18.pdb	0.988058
chr3_500kb_2.pdb	0.987943
chr3_500kb_6.pdb	0.985957
chr3_500kb_10.pdb	0.991692
chr3_500kb_20.pdb	0.990594
chr3_500kb_14.pdb	0.990660
chr3_500kb_4.pdb	0.987446
chr3_500kb_8.pdb	0.989034

3DUnicorn PDB	TMScore average
chr11_500kb_4.pdb	0.984514
chr11_500kb_10.pdb	0.984300
chr11_500kb_16.pdb	0.983752
chr11_500kb_8.pdb	0.983508
chr11_500kb_13.pdb	0.982869
chr11_500kb_12.pdb	0.976521
chr11_500kb_14.pdb	0.981787
chr11_500kb_19.pdb	0.980766
chr11_500kb_5.pdb	0.981916
chr11_500kb_11.pdb	0.979147
chr11_500kb_17.pdb	0.981465
chr11_500kb_9.pdb	0.981263
chr11_500kb_15.pdb	0.979024
chr11_500kb_7.pdb	0.980001
chr11_500kb_3.pdb	0.977934
chr11_500kb_1.pdb	0.974861
chr11_500kb_18.pdb	0.975219
chr11_500kb_6.pdb	0.975675
chr11_500kb_2.pdb	0.980590
chr11_500kb_20.pdb	0.980269

Table 2. 3DUnicorn: Average TM-score Performance Metrics for Chromosome 3 and Chromosome 11. The table presents the TM-score values averaged across pairwise comparisons, where each PDB structure is compared across each other. These metrics help evaluate structural similarities among different PDB configurations for each chromosome.

SCL PDB	TMScore average
chr3_500kb_1.pdb	0.972714
chr3_500kb_10.pdb	0.993875
chr3_500kb_11.pdb	0.989278
chr3_500kb_12.pdb	0.993925
chr3_500kb_13.pdb	0.994300
chr3_500kb_14.pdb	0.994417
chr3_500kb_15.pdb	0.994574
chr3_500kb_16.pdb	0.991536
chr3_500kb_17.pdb	0.993893
chr3_500kb_18.pdb	0.994137
chr3_500kb_19.pdb	0.993952
chr3_500kb_20.pdb	0.991185
chr3_500kb_2.pdb	0.989387
chr3_500kb_3.pdb	0.994152
chr3_500kb_4.pdb	0.993977
chr3_500kb_5.pdb	0.988761
chr3_500kb_6.pdb	0.994618
chr3_500kb_7.pdb	0.994009
chr3_500kb_8.pdb	0.994484
chr3_500kb_9.pdb	0.993820

SCL PDB	TMScore average
chr11_500kb_1.pdb	0.993237
chr11_500kb_10.pdb	0.994118
chr11_500kb_11.pdb	0.969435
chr11_500kb_12.pdb	0.994185
chr11_500kb_13.pdb	0.993802
chr11_500kb_14.pdb	0.993238
chr11_500kb_15.pdb	0.993780
chr11_500kb_16.pdb	0.994292
chr11_500kb_17.pdb	0.992302
chr11_500kb_18.pdb	0.993796
chr11_500kb_19.pdb	0.992872
chr11_500kb_20.pdb	0.992996
chr11_500kb_2.pdb	0.993463
chr11_500kb_3.pdb	0.994120
chr11_500kb_4.pdb	0.994369
chr11_500kb_5.pdb	0.993665
chr11_500kb_6.pdb	0.994047
chr11_500kb_7.pdb	0.992052
chr11_500kb_8.pdb	0.981582
chr11_500kb_9.pdb	0.993820

Table 3. SCL: Average TM-score Performance Metrics for Chromosome 3 and Chromosome 11. The table presents the TM-score values averaged across pairwise comparisons, where each PDB structure is compared across each other. These metrics help evaluate structural similarities among different PDB configurations for each chromosome.

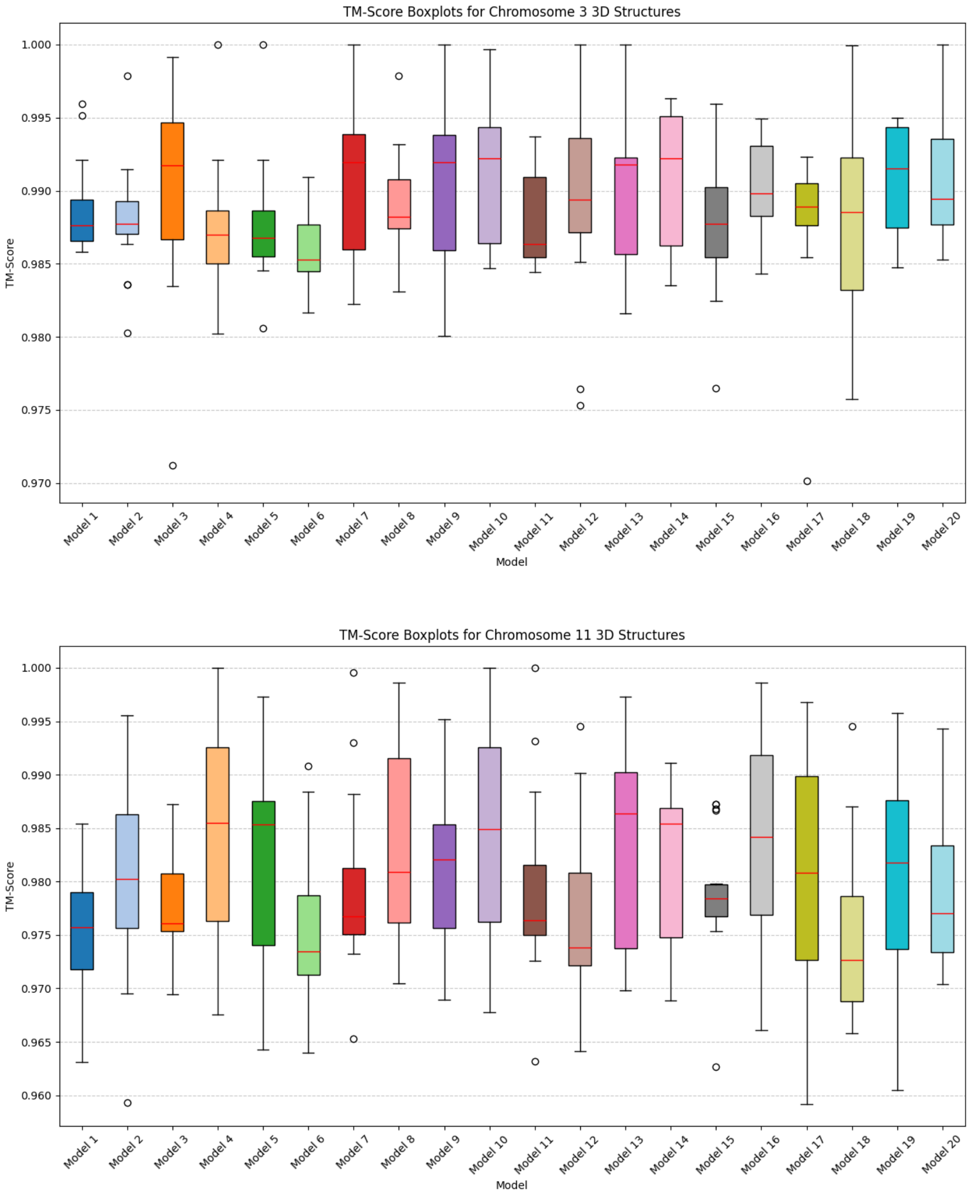

Fig. 3. Boxplot of TM-score pairwise comparisons showing structural similarity within 20 model ensembles generated by 3DUnicorn for chromosome 3(Top) and 11 (Bottom). The boxplot shows the pairwise TM-score comparisons of each structural model against all other models in the ensemble. The consistently high TM-scores indicate structural agreement across models, despite some molecular variability.

References

1. P. Hicks and O. Oluwadare. HiCARN: resolution enhancement of Hi-C data using cascading residual networks. *Bioinformatics*, 38(9):2414–2421, Apr. 2022.
2. H. Hong, S. Jiang, H. Li, G. Du, Y. Sun, H. Tao, C. Quan, C. Zhao, R. Li, W. Li, et al. Deephic: A generative adversarial network for enhancing hi-c data resolution. *PLoS computational biology*, 16(2):e1007287, 2020.
3. A. Li, G. Zeng, H. Wang, X. Li, and Z. Zhang. Dedoc2 identifies and characterizes the hierarchy and dynamics of chromatin tad-like domains in the single cells. *Advanced Science*, 10(20):2300366, 2023.
4. S. S. Rao, M. H. Huntley, N. C. Durand, E. K. Stamenova, I. D. Bochkov, J. T. Robinson, A. L. Sanborn, I. Machol, A. D. Omer, E. S. Lander, et al. A 3d map of the human genome at kilobase resolution reveals principles of chromatin looping. *Cell*, 159(7):1665–1680, 2014.
